# Quantitative Real‐Time MRI for the Assessment of Gastric Motility

**DOI:** 10.1002/jmri.70243

**Published:** 2026-02-06

**Authors:** Lydia Neubauer, Leonie Forstreuter, Fabian Winter, Fiona Mankertz, Mark O. Wielpütz, Donata S. Grajecki, Antje Steveling, Ali A. Aghdassi, Sebastian Zeißig, Matthew D. Blackledge, Dirk Voit, Jens Frahm, Susanne Schnell, Werner Weitschies, Linus Großmann

**Affiliations:** ^1^ Department of Biopharmaceutics and Pharmaceutical Technology, Institute of Pharmacy University of Greifswald Greifswald Germany; ^2^ Department of Diagnostic and Interventional Radiology University Hospital of Tuebingen Tuebingen Germany; ^3^ Department of Diagnostic Radiology and Neuroradiology University Medicine Greifswald Greifswald Germany; ^4^ Department of Internal Medicine A University Medicine Greifswald Greifswald Germany; ^5^ Division of Radiotherapy and Imaging The Institute of Cancer Research London UK; ^6^ Biomedical NMR Max Planck Institute for Multidisciplinary Sciences Göttingen Germany; ^7^ Department of Medical Physics University of Greifswald, Institute of Physics Greifswald Germany

**Keywords:** artificial intelligence, gastric emptying, motility, real‐time MRI, stomach

## Abstract

**Background:**

Current reference standards for measuring gastric emptying and motility are not considered optimal due to the time required, ionizing radiation, invasiveness, and spatial resolution.

**Purpose:**

To assess gastric motility using novel real‐time dynamic magnetic resonance imaging in combination with static measurements for gastric emptying and training of an automated deep‐learning‐based segmentation pipeline.

**Study Type:**

Prospective.

**Participants:**

The study included 36 healthy volunteers (20 female, mean 24 ± 3 years) and three patients with diagnosed Crohn's disease.

**Field Strength/Sequences:**

Participants ingested water to assess fasting motility and pineapple juice for the postprandial state. 3 T, 3D spoiled gradient echo (GRE) sequence and real‐time spoiled GRE.

**Assessment:**

Gastric emptying was measured by using the gastric volume, while motility was analyzed by tracking changes in the antrum's cross‐sectional area and applying Fast Fourier Transformation. Segmentations were performed using a trained semantic segmentation model.

**Statistical Tests:**

Linear Mixed Model with continuous dependent variables and fixed effects. Models included a random intercept for participants. Statistical significance was defined as *p* = 0.05.

**Results:**

The method enabled volumetric analysis of gastric content from 3D breath‐hold static acquisition and time‐resolved quantification of peristaltic parameters from real‐time FLASH2 imaging at high temporal resolution (here 6.24 fps). Water emptied rapidly and exponentially (*t*_1/2_ = 14.77 ± 10.55 min), while juice showed slower emptying (*t*_1/2_ = 64.24 ± 11.87 min). Contraction frequencies (fasted: 2.76 ± 0.43 cpm, fed: 2.89 ± 0.43 cpm) and velocities (fasted: 1.67 ± 0.38 mm/s, fed: 1.72 ± 0.37 mm/s) were within physiological ranges, with fasting conditions characterized by stronger occlusion compared to the fed. Measurements taken from three patients proved that the workflow could be used in a clinical context.

**Data Conclusion:**

Real‐time MRI with AI‐based analysis enabled quantitative assessment of gastric emptying and motility, revealing physiological peristaltic parameters and state‐dependent differences in occlusion.

**Evidence Level:**

2.

**Technical Efficacy:**

Stage 1.

## Plain Language Summary

Problems with stomach movement and emptying affect digestion, drug absorption, and diseases, but current tests are limited. This study tested whether fast magnetic resonance imaging combined with artificial intelligence can measure stomach emptying and movement without radiation or invasive procedures. Volunteers drank water and a calorie‐containing juice, while the stomach was imaged over time. The method measured how fast the stomach emptied and how strong and regular stomach contractions were. Results showed differences between fasting and fed states. The approach also worked in patients. This technique offers a noninvasive, quantitative way to study stomach function for research and clinical use.

## 1 Introduction

Gastric emptying is a complex physiological process essential for digestion, nutrient absorption, and oral bioavailability of drugs [1]. It is governed by coordinated gastric motility patterns that differ between fasting, postprandial, and pathological states. During fasting, the proximal stomach remains partially contracted, while the distal stomach exhibits the interdigestive migrating motor complex (IMMC) [2]. A meal interrupts the IMMC, inducing tonic relaxation in the proximal stomach and rhythmic antral contractions (~3 cpm), modulated in amplitude and propagation to facilitate digestion and controlled gastric outflow [3].

Given the sensitivity of gastric motility to disorders [4, 5, 6] and pharmaceuticals [7, 8, 9] objective and standardized tools to quantify gastric peristalsis are needed. This is especially relevant in pharmaceutical development, where peristaltic changes can mainly influence absorption of compounds with pH‐dependent solubility, narrow absorption windows, or modified‐release profiles [9].

Several techniques have been established to assess gastric motility or its surrogate, gastric emptying, including scintigraphy, manometry, isotope breath tests, and the gastric barostat [10]. While scintigraphy remains the reference standard for gastric emptying, it lacks spatial and functional detail and involves ionizing radiation. Manometry and barostat‐based methods provide detailed physiological information but are invasive, time‐consuming, and poorly suited for repeated measurements in healthy volunteers. In contrast, MRI has emerged as a non‐invasive alternative capable of assessing gastric volume, emptying, and wall motion simultaneously [11, 12, 13]. Despite these advantages, quantitative analysis of gastric peristalsis using MRI remains methodologically challenging. Many studies rely on manual, frame‐by‐frame measurements and evaluate only a limited number of slices due to the high analysis burden [13, 14, 15], resulting in limited standardization and susceptibility to interobserver variability. Although commercial solutions such as Entrolytics (Motilent) enable robust analysis of gastrointestinal motility, primarily targeting small‐bowel dynamics, and research approaches such as spatiotemporal motility mapping have been applied to the stomach, fully automated, open‐source tools for detailed antral peristaltic assessment remain scarce in clinical practice [16, 17].

The purpose of this study was to develop and validate a standardized MRI protocol with an automated pipeline for quantitative assessment of gastric emptying and antral motility in fasted and fed states. Real‐time imaging used the radial FLASH2 gradient‐echo sequence [18], which enables high temporal resolution and permits T1‐weighted stomach content tagging (e.g., manganese), unlike balanced SSFP approaches commonly used for gastrointestinal motility imaging [12, 19, 20].

## 2 Materials and Methods

### 2.1 Participants

The prospective study protocol was approved by the Ethics Committee of the University Medicine Greifswald, Germany (Reg.‐Nr. BB 072/24b, DRKS00035519). The study was conducted in accordance with the latest version of the Declaration of Helsinki. All participants provided written informed consent.

Thirty‐six healthy participants were recruited. Inclusion criteria were age between 18 and 55 years, BMI between 18 and 30 kg/m^2^ and health assessed by the investigating physician as clinically unremarkable. Exclusion criteria were any absolute or relative contraindications to MRI.

To test the application of the measurement methodology on patients, three patients with diagnosed Crohn's disease and clinically suspected gastroparesis, which needed to be verified, were recruited from the Department of Internal Medicine at the University Medicine Greifswald, Germany.

### 2.2 Study Protocol

Alcohol consumption had to be stopped at least 24 h before the study day. Participants were not allowed to eat for at least 10 h; high‐calorie drinks were to be avoided for 3 h and water for 90 min prior to the study. The consumption of caffeinated beverages was not permitted.

**FIGURE 1:**
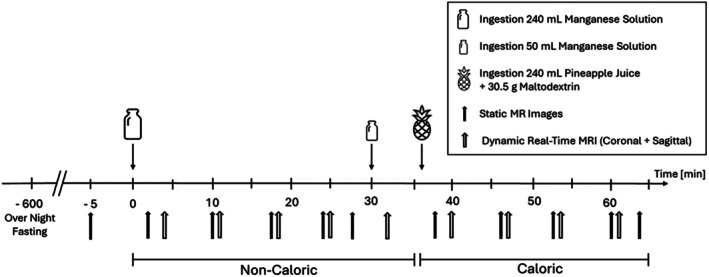
Study procedure: Timeline with interventions explained in legend.

As can be seen in Figure 1, participants' fasting states were first verified (*t* = −5 min). Then, they consumed 240 mL of manganese‐labeled water in an upright position. At *t* = 30 min and *t* = 36 min, participants were removed from the scanner to ingest an additional 50 mL of the manganese labeled water and 240 mL of calorie‐enriched pineapple juice, respectively, to establish fed conditions. At time points *t* = 2, 10, 17, 24, 28, 38, 46, 53, 60, 64 min, static MR images (Volumetric Interpolated Breath‐hold Examination [VIBE]) were acquired. Dynamic real‐time MRI (rtMRI) were acquired at *t* = 4, 11, 18, 25, 32, 40, 47, 54, 61 min. The timing was chosen to strike a balance between achieving the closest possible control and the time required to reconstruct the images/videos.

### 2.3 Composition of the Manganese Labeled Water

The non‐lanthanoid and food additive manganese(II) gluconate dihydrate (Dr. Paul Lohmann GmbH und Co. Germany) was used for T1‐contrast [21]. The participants received a total of 290 mL (circa 15°C) of manganese‐labeled water (0.73 g per 1 L tap water), resulting in a manganese dose (Mn^2+^) of 24.2 mg.

### 2.4 Composition of the Calorie‐Enriched Pineapple Juice

To avoid additional exogenous contrast agents, naturally manganese‐rich pineapple juice (Refresco Deutschland GmbH, Germany) was used. To reliably induce a postprandial gastric motor pattern, 30.5 g maltodextrin (Nutricia GmbH, Germany) was added to 240 mL pineapple juice, resulting in a caloric load of 250 kcal.

### 2.5 MRI Acquisition

MRI were acquired on a clinical 3 T MRI scanner (MAGNETOM Vida, Siemens Healthineers, Germany) with an 18‐channel body and 32‐channel spine coil in supine position. T1‐weighted spoiled GRE sequences (VIBE) were used to visualize the abdomen in the coronal and transverse planes. The transversal images at each time point (Figure 1 and Study Protocol) were used to determine the gastric content volume (GCV) over time. The coronal VIBE protocol used a TR = 5.99 ms, TE = 2.46 ms, slice thickness of 4 mm, 60 slices, flip angle = 30°, matrix 320 × 161, FOV 550 × 395 mm and a 20 s inspiratory breath‐hold. Transverse VIBE used matched parameters but with TR = 6.03 ms, matrix size 448 × 176 and FOV 550 × 309 mm. To analyze the peristalsis, T1‐weighted radial real‐time spoiled GRE sequences (FLASH2) were acquired in the coronal and sagittal planes. Both FLASH2 sequences used a TR = 3.08 ms and TE = 1.9 ms, slice thickness of 5 mm, and three interleaved slices with a flip angle of 20°, matrix 140 × 140, and FOV 200 × 200 mm. Each frame consisted of 13 radial spokes, resulting in an effective frame rate of 6.24 fps including the time for CHESS fat saturation before each frame. FLASH2 data were acquired for 180 s during free breathing.

FLASH2 slice positioning was guided by transverse VIBE images (at *t* = 2 min in fasting; *t* = 38 min postprandial states) and kept constant between these time points. Three interleaved slices were acquired at fixed spacing. Because VIBE was acquired under breath‐hold, coronal FLASH2 slices were placed at the inferior antral margin to compensate for the cranial shift during free breathing. For sagittal FLASH2, slices were positioned relative to the antrum/pylorus: slice 1 anterior to the pylorus, slice 2 mid‐antrum, slice 3 near the lesser curvature. Exemplary placement of the slices can be found in the Supporting Information (Figure S1). The distance factor (DF) was adapted individually (1.5–3.5), yielding inter‐slice distances of 7.5–17.5 mm. Recordings lasted 1.5 min at *t* = 25, 32, and 61 min and 3 min at all other time points.

### 2.6 MRI Acquisition of Patients

To test the application of the measurement methodology on patients, only a postprandial (*t* = 0 min ingestion of calorie‐enriched pineapple juice) two‐point determination of stomach volume (*t* = 11, 46 min) and peristalsis (*t* = 18, 38 min) were performed for a patient‐friendly setup. At these timepoints, the used sequences were the same as in the main study.

### 2.7 Training of Artificial Intelligence

Gastric segmentation used nnU‐Net (nnU‐Net v2.0) [22, 23] with two models: *Volyntra* (transverse volumetry) and *Motiqva* (sagittal real‐time). Training details are provided in the Supporting Information.

### 2.8 Inference Pipeline and Manual Review

Inference was performed with a custom Python 3.12 script executing *Volyntra* and *Motiqva* after DICOM‐to‐NRRD conversion. The script automated model inference, postprocessing, and output generation. For each subject and time point, the script produced the following outputs:

- .nnrd files for each transverse VIBE series and sagittal FLASH2‐sequence;
- a .csv file for each subject containing the computed gastric content volumes (*Volyntra*) or segmented stomach areas (*Motiqva*) over time;
- an .mp4 video showing the MR sequence overlaid with the generated segmentation masks.

This video output enabled fast visual inspection of segmentation quality in a human‐in‐the‐loop review process without iterative retraining (annotation and revision by LN, 2 years expertise, revision by LG, 5 years expertise and WW, 35 years of expertise in gastrointestinal imaging). In cases where obvious segmentation errors were identified by all three observers, the corresponding .nrrd image and label were imported into 3D Slicer (version 5.8.1, The Slicer Community, USA) for manual correction [24]. After editing, the corrected mask was again reviewed and afterwards reintegrated into the analysis pipeline.

If all three observers independently decided that a stomach cannot be clearly identified, it was designated as a dropout.

### 2.9 Volume Evaluation

Gastric content volume was computed by voxel counting in the segmented transverse VIBE masks and conversion to mL using voxel dimensions. Volumetry captured only T1‐bright manganese‐enhanced content; baseline values therefore reflect absence of labeled content rather than absolute fasting gastric volume. Postprandial emptying rates were calculated as the linear slope of the decrease in gastric content volume over time as emptying rate = Δ*V*/Δ*t*. For better comparison, the emptying half‐life was also calculated with *t*_1/2_ = ln (2)/*k* for first‐order kinetics and with *t*_1/2_ = *c*_0_/2*k* for zero‐order kinetics.

### 2.10 Motility Evaluation

To describe gastric peristalsis, quantitative parameters such as frequency, wave velocity, and occlusion were extracted from segmented area‐time curves obtained from sagittal FLASH2 sequences. For each subject and each of the nine measurement time points, area signals were analyzed separately for the three slices. For display only, area‐time curves were smoothed with a Savitzky–Golay filter (window 59 frames, order 1); all frequency analyses used raw signals.

Dominant contraction frequencies were identified via Fast Fourier Transformation (FFT) within the band of 0.03–0.07 Hz (1.8–4.2 cpm). The frequency band was applied as a bandpass filter to suppress irrelevant signal components. Frequencies with a dominance factor ≥ 2.0, defined as the ratio of peak amplitude to the mean amplitude within the band, were considered valid indicators of peristaltic activity. Mean values for wave frequency were calculated only at time points in which at least two out of three sagittal slices exhibited a dominance factor ≥ 2.0, indicating a sufficiently robust signal for frequency detection. As wave velocity was calculated based on frequency, it was only calculated at time points for which frequency was calculated.

Occlusion was defined as the relative reduction in cross‐sectional area during a contraction, normalized to a reference area. For every subject and each measurement time point of the study protocol and for each of the three sagittal slices, all frames of the corresponding real‐time sequence were analyzed separately. The reference area was calculated per slice and per measurement time point as the median of the top 10% largest areas within this sequence. It was calculated as:

(1) $$\;\text{Occlusion}\;\left[\%\right]=100\times \left(1-\frac{\text{Area}}{\text{Median of}\;\mathrm{top}\;10\%\;\text{Areas}}\right)$$

with all negative values clipped to 0%, as occlusion cannot be smaller than the fully relaxed state. Mean occlusion values were computed per time point and slice to estimate contractile strength, with complete antral closure corresponding to 100% occlusion.

Propagation was estimated using the subject‐specific inter‐slice distance (7.5–17.5 mm) and the effective sampling distance between slice 1 and slice 3:

(2) $$\begin{array}{cc} \text{Propagation Distance}\;\left[\mathrm{mm}\right]= & 3\times \text{Slice Thickness}\\ & +2\times \text{Inter}-\text{slice Distance} \end{array}$$

To obtain a spatio‐temporal metric, an apparent propagation speed was computed as:

(3) $$\begin{array}{cc} \text{Apparent Propagation Speed}\;\left[\frac{\mathrm{mm}}{s}\right]= & \left(\frac{\text{Frequency}\;\left[\mathrm{cpm}\right]}{60}\right)\\ & \times \text{Propagation Distance}\;\left[\mathrm{mm}\right] \end{array}$$

This value reflects an apparent (rather than true phase) propagation speed, since the peristaltic wave spans multiple slices and does not exhibit a sharply localized spatial peak. To assess the validity of this geometric approximation, a time‐delay cross‐correlation analysis between slices was performed for a representative dataset, yielding propagation delays and speed estimates within the same physiological range (see Supporting Information).

**FIGURE 2:**
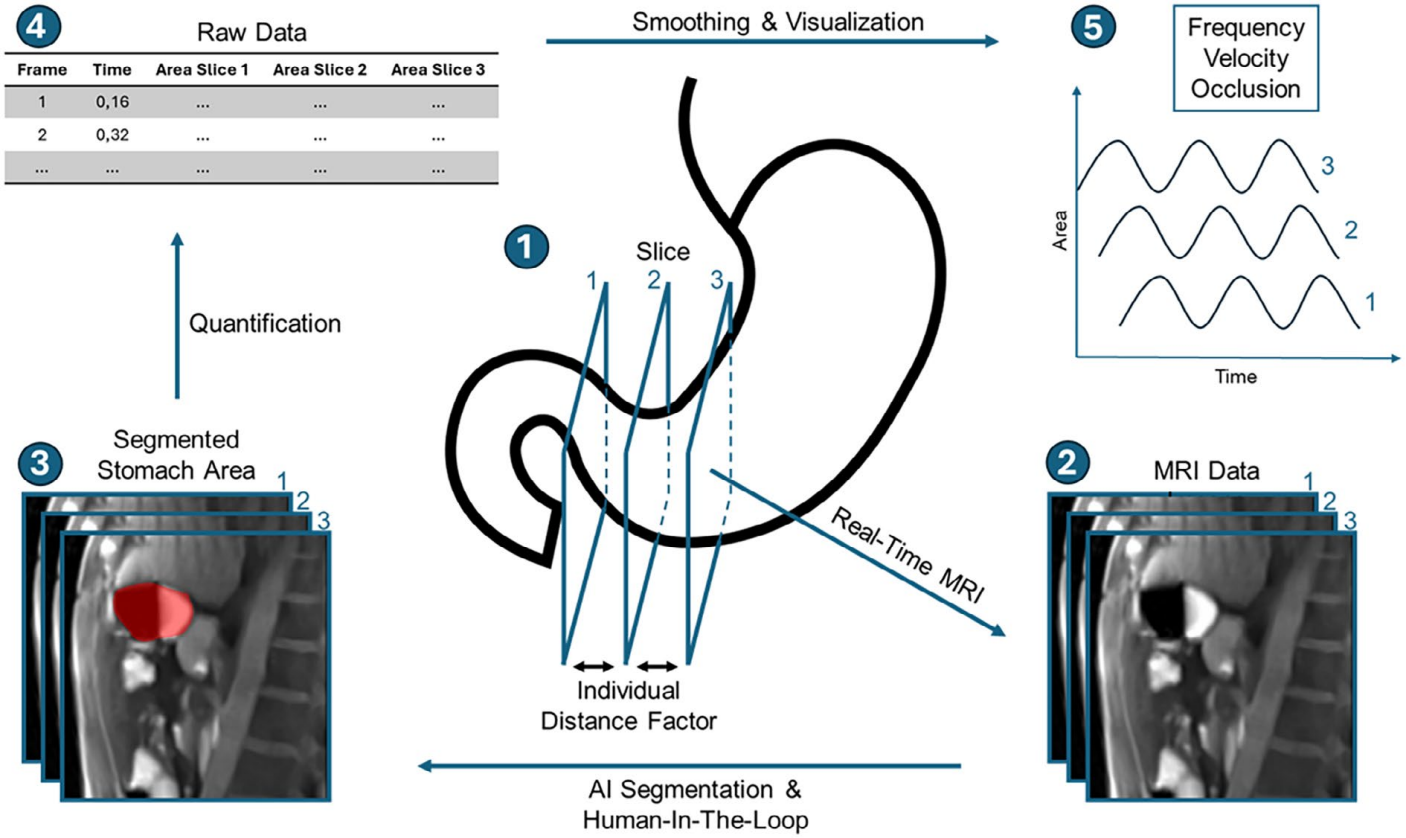
Illustration of the methodological procedure for requiring motility evaluation: 1: DF and slice positioning, 2: Requiring MRI data, 3: AI segmentation and manual correction (human‐in‐the‐loop), 4: Quantification of raw area data, 5: Calculation, smoothing and visualization of frequency, velocity, and occlusion.

The full workflow is illustrated in Figure 2.

### 2.11 Statistics

To examine the effects of emptying water and pineapple juice on physiological responses, three separate linear mixed‐effects models (LMMs) using *jamovi* (version 2.6, The jamovi project, Australia) with the GAMLj plugin were conducted [25]. Each model used a continuous dependent variable and included the fixed effects *condition* (fasted/water vs. fed/juice), *timepoint* and their interaction. For the occlusion model, *slice* (1‐3) and all two‐ and three‐way interactions (*slice × timepoint × condition*) were included. All models included a random intercept for participants to account for repeated measurements. Degrees of freedom were estimated using the Satterthwaite approximation, and residual normality was assessed using Kolmogorov–Smirnov and Shapiro–Wilk tests. Statistical significance was defined as *p* = 0.05.

## 3 Results

Thirty‐six healthy participants (20 female, mean 24 ± 3 years, BMI 22.3 ± 2.7 kg/m^2^) were included and completed the prospective study, with no reports of adverse events. One dropout occurred in the automatic segmentation of *Motiqva* because the stomach could not be clearly identified in the sagittal plane. Gastric emptying and gastric peristalsis were successfully determined under interleaved slice acquisition of three slices with a framerate of 6.24 images per second, resulting in up to 3375 (and 1650 for 1.5 min) images per measuring time point.

Three patients (patient 1: male, 69 years, BMI 21.4 kg/m^2^, patient 2: female, 46 years, BMI 18.3 kg/m^2^, patient 3: male, 27 years, BMI 23.1 kg/m^2^) also completed MRI in a patient‐friendly shortened protocol to check for a clinically suspected gastroparesis.

### 3.1 Human‐in‐the‐Loop: Manual Correction

To ensure the quality and anatomical accuracy of the automated segmentations, all generated .mp4 videos were reviewed in a human‐in‐the‐loop workflow. Of a total of 396 VIBE sequences, 21 sequences (5.3%) of 14 participants were corrected. Of a total of 945 FLASH2‐sequences, 250 (26.5%) of 29 participants were corrected. Notably, segmentation performance was consistently higher in regard to the corrected frames in the transverse VIBE images (*Volyntra*) compared to the sagittal FLASH2‐sequences (*Motiqva*), where motion artifacts and variable stomach shapes led to more frequent inaccuracies. Segmentation errors included incomplete gastric wall outlines, mislabeled structures, masks extending beyond anatomy, and missing segmentations. Details of the AI dataset, segmentation results and computation time can be found in the Supporting Information.

### 3.2 Gastric Content Volume

**FIGURE 3:**
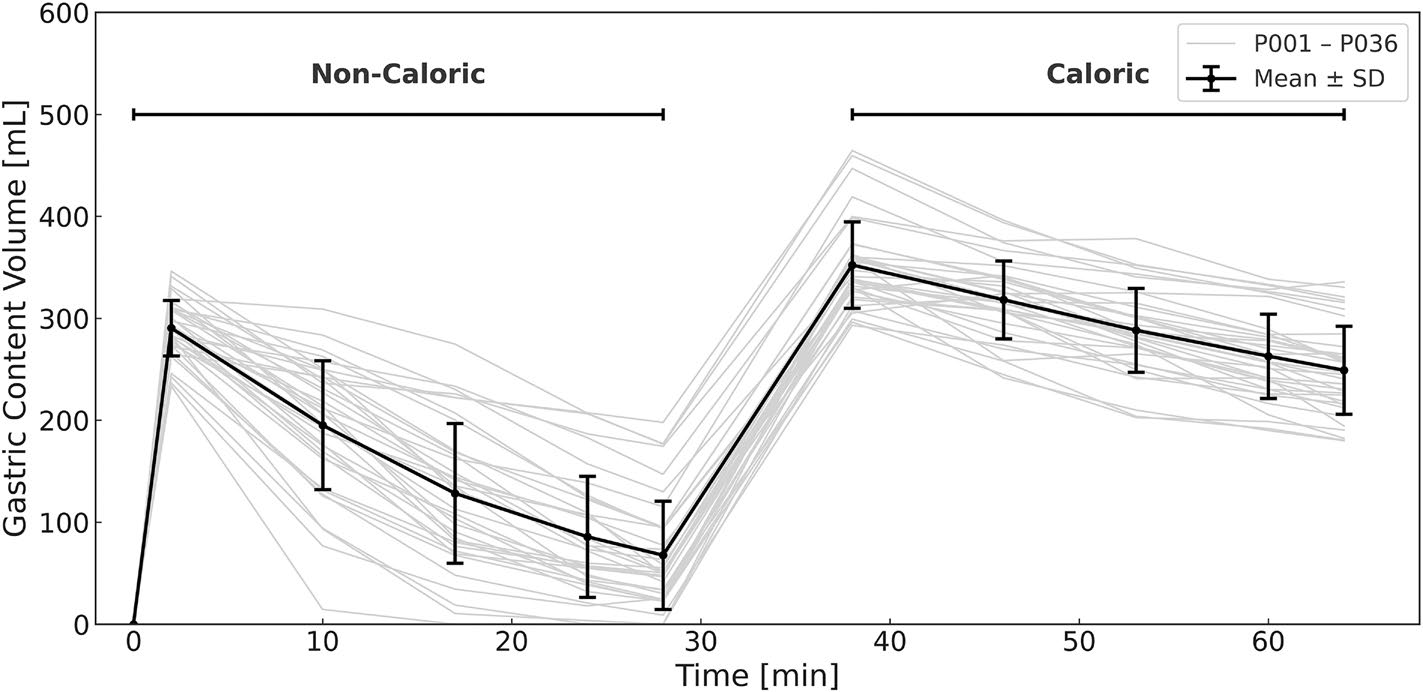
Gastric content volume (GCV) over 64 min, individual participants shown in light gray, mean ± SD shown in black.

Figure 3 shows the gastric content volume (GCV) over time for all 36 participants, quantified using *Volyntra* segmentations. Subject individual GCV can be seen in the Supporting Information (Figure S2). After ingestion of the manganese‐labeled water, GCV decreased progressively during the fasting phase, with a mean peak volume of 290.38 ± 27.23 mL. Following intake of the calorie‐enriched pineapple juice, GCV increased again rapidly to a mean postprandial peak of 352.3 ± 42.4 mL. Average emptying rates were calculated only for the postprandial interval (38–64 min) and amounted to 4.0 ± 1.1 mL/min for pineapple juice (38–64 min). Corresponding emptying half‐lives were *t*_1/2_ = 14.77 ± 10.55 min for water and *t*_1/2_ = 64.24 ± 11.87 min for the pineapple juice.

### 3.3 Gastric Motility

**FIGURE 4:**
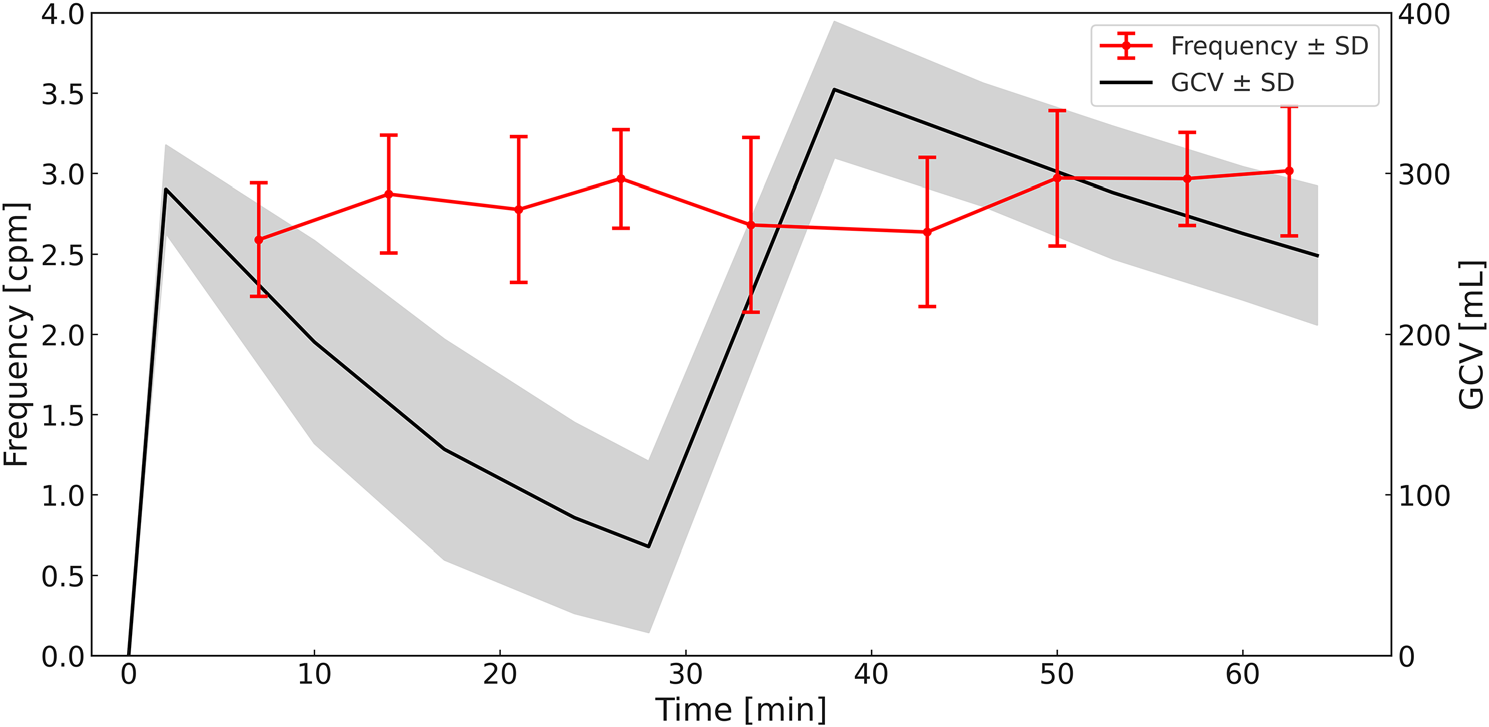
Mean frequency (red) ± SD and gastric content volume (GCV) (black) ± SD over 64 min.

Mean gastric contraction frequency ranged from 2.59 to 3.02 cpm (Figure 4) across the nine measurement time points with standard deviations between 0.29 and 0.54 cpm. Contraction frequency in the fasted state was slightly, though significantly and consistently, lower than in the postprandial state, with a modest increase observed over time during both the 7–33.5 and 43–62.5 min intervals. The interaction was not significant (*p* = 0.671). Marginal and conditional *R* ^2^ were 0.122 and 0.357, respectively.

**FIGURE 5:**
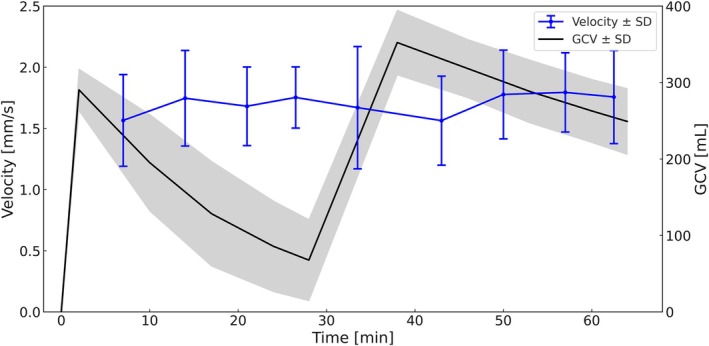
Mean velocity (blue) ± SD and gastric content volume (GCV) (black) ± SD over 64 min.

Wave velocity (Figure 5) ranged from 1.56 to 1.79 mm/s, with standard deviations between 0.25 and 0.50 mm/s. Velocity was significantly lower in the fasted state and increased over time in both conditions, again without significant interaction. Marginal and conditional *R* ^2^ were 0.055 and 0.676, respectively.

**FIGURE 6:**
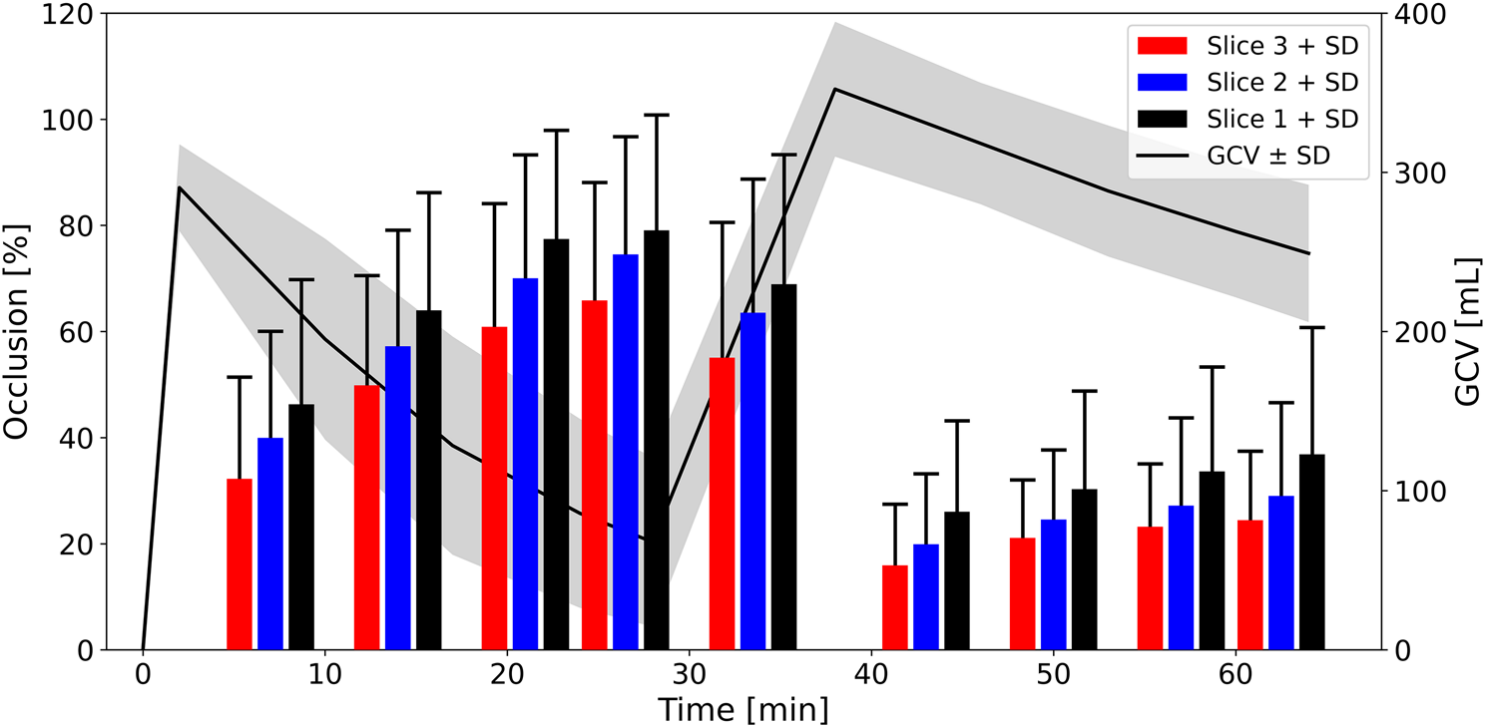
Mean occlusion + SD of slice 1 (black), 2 (blue), and 3 (red) and gastric content volume (GCV) (black) ± SD over 64 min.

In Figure 6 occlusion increased during the fasted state (7–33.5 min), peaking at 26.5 min (e.g., slice 1: 79.1% ± 21.8%) and dropped markedly after juice intake. Occlusion was lowest in the lesser curvature (slice 3) and increased towards the pylorus (slice 1). Significant main effects of slice and time point were observed, with a significant condition × time interaction. Marginal and conditional *R* ^2^ were 0.497 and 0.657, respectively.

**FIGURE 7:**
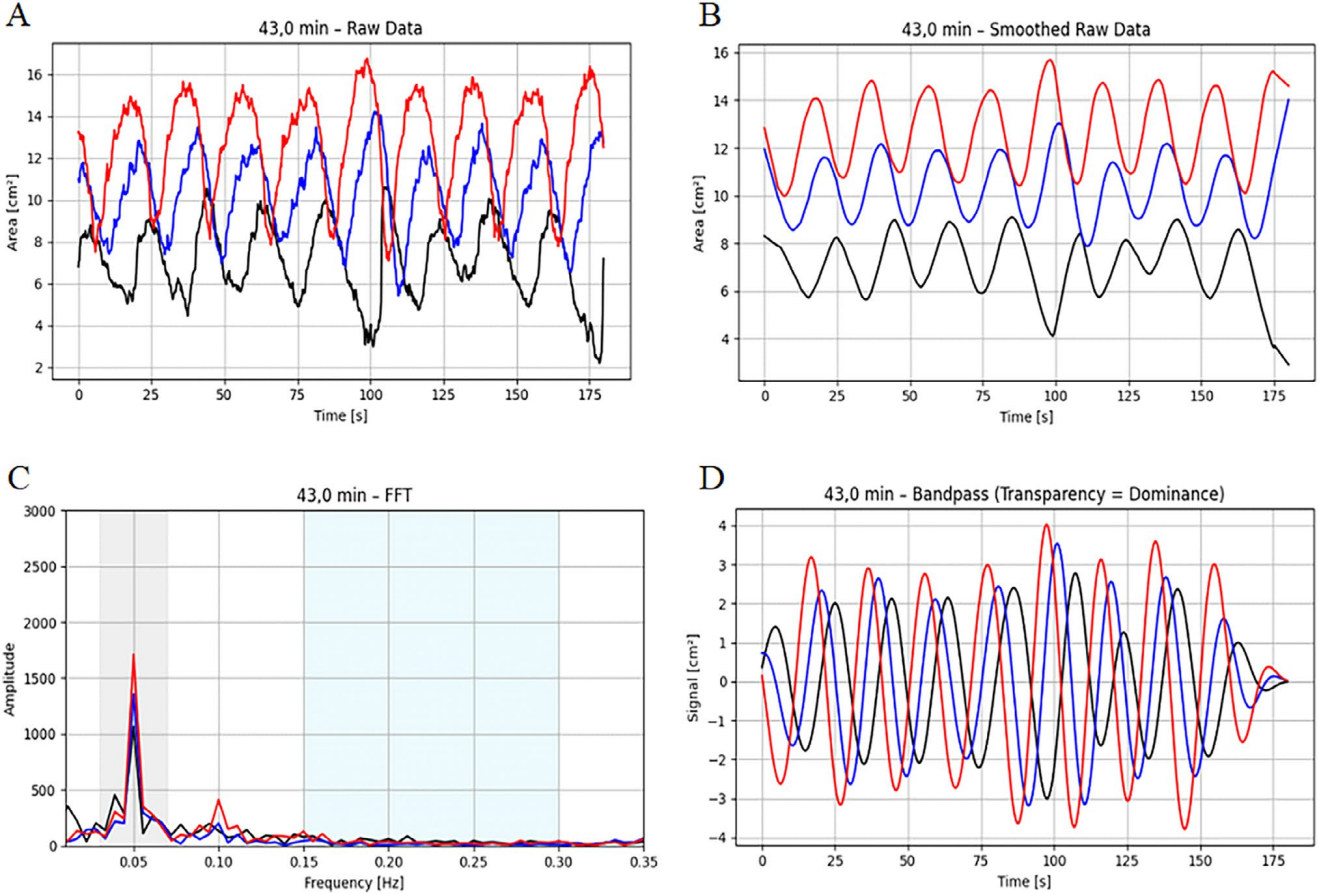
Visualization of raw data (A), smoothed data (B), FFT, gray = 0.03–0.07 Hz (frequency band), blue = 0.15–0.30 Hz (breathing band) (C), and bandpass (D) at 43 min, P021, slice 1 = black, slice 2 = blue, slice 3 = red.

**FIGURE 8:**
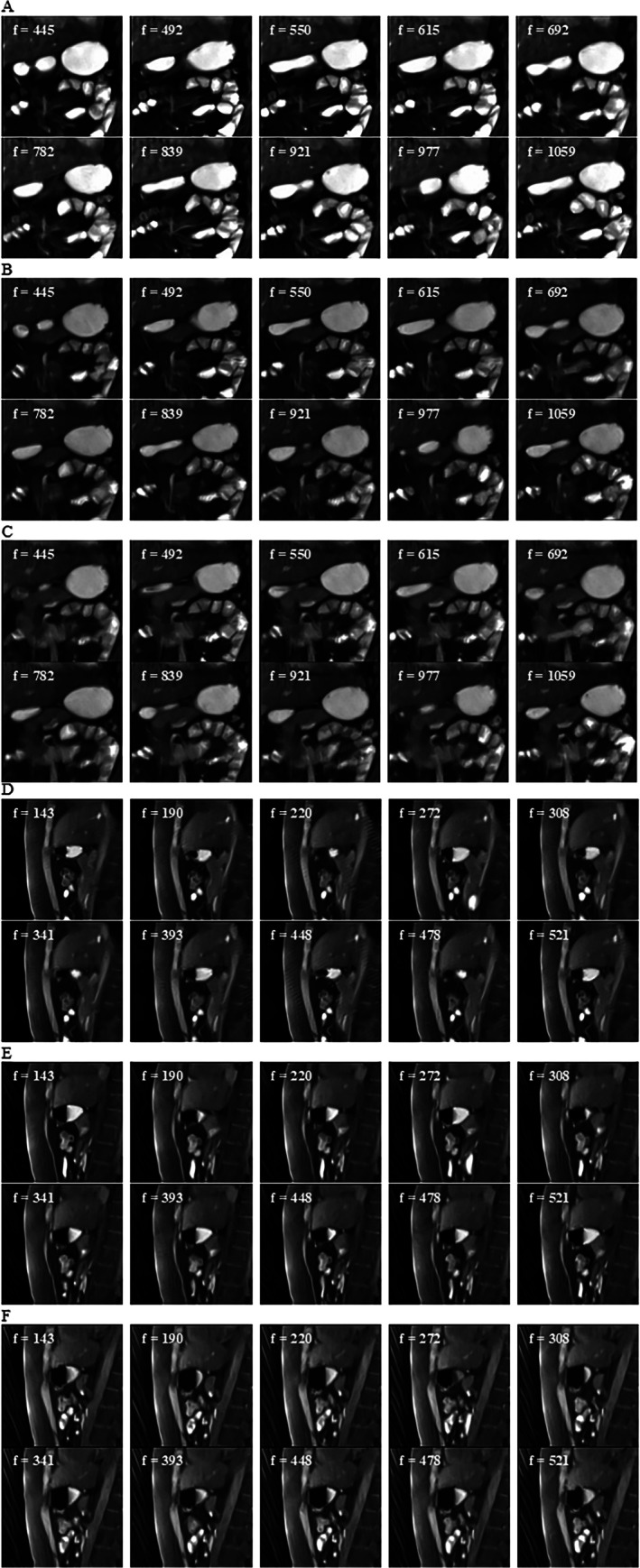
Selection of snapshots (f = frame) of the coronal stomach in a FLASH2‐sequence at 40 min (A = slice 1, B = slice 2, C = slice 3), participant P021 and selection of snapshots (f = frame) of the sagittal stomach in a FLASH2‐sequence at 43 min (D = slice 1, E = slice 2, F = slice 3), participant P021.

An exemplary segmentation and signal analysis is shown in Figure 7, demonstrating clear rhythmic contractions across all three slices with dominant FFT peaks in the expected gastric frequency range and no interference from respiratory frequencies. Spatial confirmation of wave propagation is illustrated by selected snapshots in Figure 8.

**FIGURE 9:**
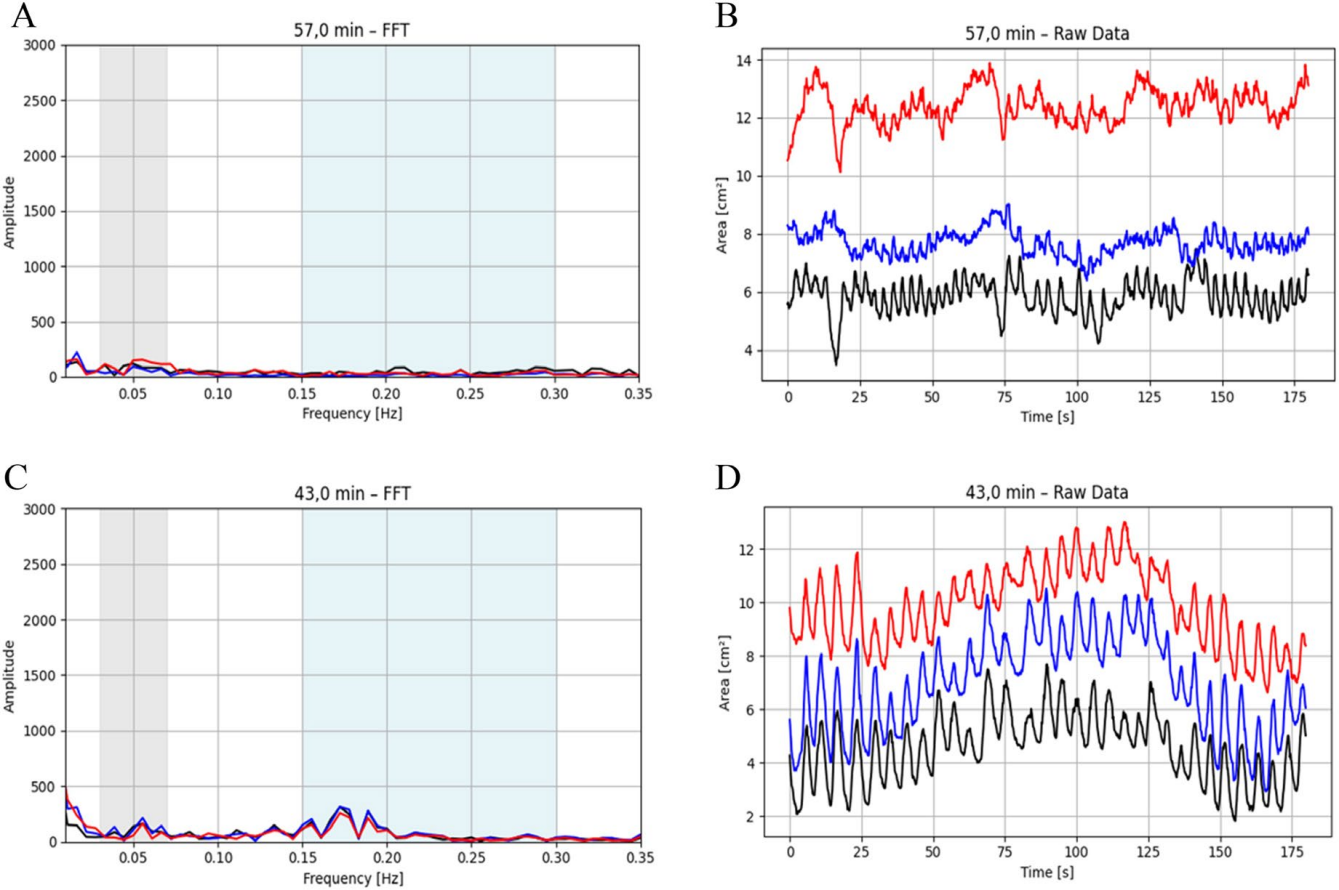
Visualization of undefined peristalsis: FFT: gray = 0.03–0.07 Hz (frequency band), blue = 0.15–0.30 Hz (breathing band) (A, C) and raw data (B, D), P001 (A, B) at 57 min and P008 (C, D) at 43 min, slice 1 = black, slice 2 = blue, slice 3 = red.

**FIGURE 10:**
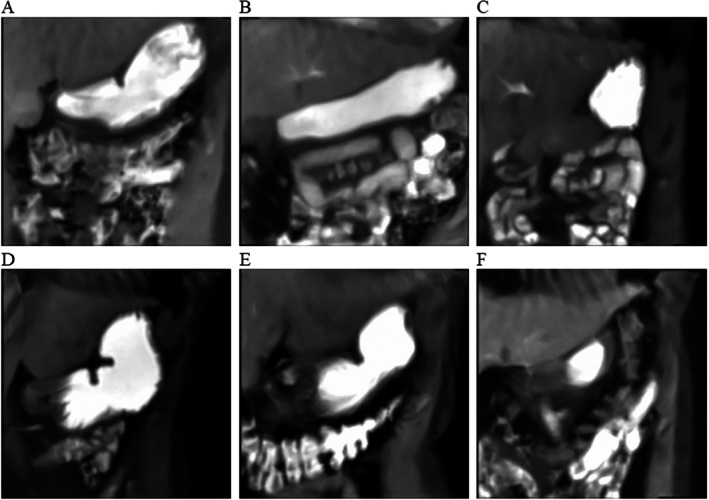
Snapshots of coronal stomachs: A = P022, 40 min, slice 1; B = P006, 40 min, slice 3; C = P030, 18 min, slice 1; D = P025, 11 min, slice 1; E = P009, 4 min, slice 1; F = P026, 47 min, slice 3.

Examples of non‐quantifiable peristalsis are shown in Figure 9, where either irregular low‐amplitude signals or dominant respiratory oscillations prevented reliable extraction of peristaltic parameters. Figure 10 further illustrates pronounced inter‐individual anatomical variability of the antrum in coronal orientation, highlighting challenges for peristaltic quantification in some participants.

The numbers of participants included in each timepoint for the calculation of frequency and velocity are reported in the Supporting Information (Table S1).

### 3.4 Patients

Gastric peristalsis could be successfully measured in all three patients. Motility was comparable to the healthy study population with frequencies around 3 cpm and velocities of circa 1.7 mm/s with stronger occlusions towards the pylorus. However, GCV values stagnated or even increased over the observation period of 45 min at about 250 mL. The detailed results for volumes and motility data can be found in the Supporting Information (Table S2).

## 4 Discussion

This study demonstrates the feasibility of combining high‐temporal‐resolution real‐time MRI with automated analysis to quantify gastric emptying and antral motility in fasting and postprandial states. *Volyntra* provided time‐resolved gastric content volumes, while *Motiqva* enabled extraction of peristaltic parameters from sagittal FLASH2 sequences. Feasibility was additionally explored in three Crohn's disease patients with suspected gastroparesis.

Gastric emptying profiles clearly differentiated between water and nutrient‐containing liquid. Water emptied rapidly with an exponential decay (*t*_1/2_ ≈ 15 min), consistent with first‐order kinetics and literature values between 8 and 18 min [26, 27]. In contrast, the pineapple juice showed a slower and more linear decrease in volume, characteristic of zero‐order emptying governed by regulatory feedback mechanisms [28]. This aligns with established physiological principles, where non‐caloric liquids primarily empty according to pressure gradients but still evoke gastric mechanosensory responses and transient motor adjustment, while caloric beverages trigger pyloric control and neurohormonal responses that modulate emptying [29, 30]. Interindividual variability was more pronounced during water emptying, in line with known variability of fasting gastric tone and compliance, whereas fed‐state motility is more tightly regulated [31, 32]. The estimated caloric emptying rates after juice ingestion fell within physiologically expected ranges (2–4 kcal/min) [33, 34], supporting the validity of the approach, though dilution by gastric secretions must be considered.

For peristaltic measurements, extracted parameters—contraction frequency, velocity and occlusion—reflect key physiological aspects of gastric motility: intrinsic pacemaker activity of the stomach, neuromuscular coordination and the mechanical efficacy of the contractions [35]. The mean contraction frequency was approximately 2.8 cpm, which falls within the physiological range of 2.5–3.5 cpm reported in the literature for healthy individuals [15, 36]. Similarly, the mean wave velocity of 1.7 mm/s was in line with previously published values of 2–4 mm/s, depending on gastric region and content [15, 36]. Notably, in a prior study a slowing of peristaltic activity in non‐upright positions, such as the upside‐down posture, is shown [37]. This finding may extend to the supine position used in our study and could partially explain the observed minimal lower frequencies or slowed propagation in some cases.

While this study aimed to compare fasting and postprandial peristaltic patterns, the statistical differences in contraction frequency and wave velocity were significant, although the absolute values remained close. The 30 min fasting window was insufficient to capture distinct IMMC phases (typically 90–120 min). Moreover, peristaltic analysis requires a non‐collapsed stomach, limiting quantification at near‐empty time points.

Occlusion was higher during fasting than after meal ingestion, consistent with increased gastric tone and more forceful contractions in the fasted state [38]. For occlusion, a direct quantitative comparison with literature is limited due to methodological variability in how this parameter is defined and measured, but the relative trend in our data supports established physiological concepts.

Automating segmentation and peristalsis extraction substantially reduced manual workload and interobserver variability compared to traditional frame‐by‐frame annotation approaches. The implemented human‐in‐the‐loop review ensured robustness without introducing iterative retraining cycles. Together, this workflow enables standardized, non‐invasive assessment of gastric volume and motility, extending beyond surrogate measures such as scintigraphic emptying alone.

While coronal FLASH2 acquisitions were performed in this study, they were ultimately not used for quantitative peristaltic evaluation. This was due to the considerable anatomical variability of the stomach, which often prevents consistent imaging of the entire antrum within a single slice across different participants. As shown in the results, some stomachs do not allow a peristaltic wave to be tracked based on the anatomical features. Sagittal imaging, however, provided a full visualization of the antrum and pylorus, as the antral region typically appears nearly circular in this plane.

Because validated decision criteria for “regular” versus “irregular” peristalsis in FLASH2 are lacking, interpretation currently requires reference data from large healthy cohorts. As each participant was measured once, intra‐subject variability could not be assessed; future studies should address day‐to‐day and hormonal influences and enable comparisons with patient cohorts [31, 39, 40].

The framework may support research and clinical evaluation of motility‐modifying interventions and functional gastrointestinal disorders, complementing established techniques with non‐invasive, quantitative MRI readouts [41, 42]. Future studies should investigate the clinical applicability of this method in patient populations in more depth and assess its diagnostic value in comparison with established techniques. To obtain a first look into a small patient cohort, we measured three individuals with known Crohn's disease and possible gastroparesis. With a simplified protocol, we were able to visualize their gastric motility and emptying, revealing active motility but altered gastric emptying for all patients. This was most likely caused by a Crohn's disease condition in their small intestine and a constant backflow of chyme into the stomach and therefore a stagnation of gastric content volume over a period of circa 45 min, which normally cannot be observed in a healthy population [43]. Due to the small sample size and the focus on a healthy study group, we decided not to present average values or conduct an in‐depth analysis of the clinical picture.

In this context, an important opportunity lies in adapting the proposed framework to cine‐MRI sequences, which are already integrated into routine clinical imaging protocols, especially for heart disease [44] but also for the abdomen [45]. Depending on the scanner and protocol, abdominal cine‐MRI typically achieves temporal resolutions of 2–4 fps [46, 47], which should be sufficient to resolve physiological peristaltic frequencies.

However, transferring the *Motiqva/Volyntra* framework to other sequences and test meals would require sequence specific (re)training of the segmentation network [48] and sufficient antral/pyloric coverage (multi‐slice/stack acquisition). Although 3D real‐time approaches have been demonstrated for other GRE‐based methods (e.g., van Harten et al.), the FLASH2 sequence used in our protocol is a 2D radial real‐time implementation [49]. Multi‐slice or stack strategies with lower frame rates may therefore be the most feasible near‐term option [49]. Integration into radiology software (e.g., PACS) would further support clinical adoption.

If these technical challenges can be addressed, this framework could pave the way towards clinically scalable, non‐invasive gastric motility assessment using already available imaging infrastructure. This would lower the barrier to implementation, reduce examination time, and bring real‐time motility assessment a decisive step closer to clinical routine.

### 4.1 Limitations

Several limitations should be acknowledged. Reliable peristaltic analysis requires sufficient gastric filling. Near‐complete gastric collapse prevented quantitative evaluation at some time points. This behavior is expected, as the segmentation model relies on T1‐weighted signal from manganese‐containing fluid to delineate the gastric content. Slice positioning and field‐of‐view selection critically influence signal quality, particularly near the pylorus, where small diameters limit measurable area changes. Consequently, frequency and velocity could not be extracted for all participants at all time points.

Additionally, this was a single‐center study with a relatively small cohort, using a single MRI vendor and field strength. These factors may limit generalizability and warrant validation in multi‐center, multi‐vendor settings.

## 5 Conclusion

This study demonstrated the feasibility of abdominal real‐time FLASH2 together with an artificial intelligence‐guided post‐processing pipeline for quantitative assessment of gastric motility. By combining it with established GRE sequences, it allowed measurement of gastric volume and motility, capturing differences in emptying kinetics and peristaltic parameters in one protocol. Despite limitations like gastric filling and interindividual variability, the approach reduces manual effort and interobserver variability, providing a potentially scalable tool for research and clinical use. Importantly, it lays the groundwork for building normative datasets that could support future diagnostic use in functional gastrointestinal disorders as well as understanding gastric contribution in drug absorption.

## Funding

The University of Greifswald received funding from the German Research Foundation (DFG, INST 292/155‐1 FUGG). This study was also supported by Health.AI Pomerania (grant INT0100008).

## Supporting information


**Data S1:** jmri70243‐sup‐0001‐DataS1.zip.
