# Supplementary material for: Quantitative Real‐Time MRI for the Assessment of Gastric Motility
Source: J Magn Reson Imaging. 2026 Feb 6;63(5):1482–95. doi: 10.1002/jmri.70243 (PMC13066535; doi:10.1002/jmri.70243)
Supplement: Supplementary file 1 — Data S1: jmri70243‐sup‐0001‐DataS1.zip. [file JMRI-63-1482-s001.zip › video captions-JMRI_70243__author.docx]

**Captions for submitted zipped videos:**

**In zipped folder: videos_P001**

Name of file: P001_cor_54_1

Caption: Coronal real-time spoiled GRE (FLASH2) of the stomach from participant P001 at t = 54 min. Slice 1 of 3 in 6.24 fps.

Name of file: P001_cor_54_2

Caption: Coronal real-time spoiled GRE (FLASH2) of the stomach from participant P001 at t = 54 min. Slice 2 of 3 in 6.24 fps.

Name of file: P001_cor_54_3

Caption: Coronal real-time spoiled GRE (FLASH2) of the stomach from participant P001 at t = 54 min. Slice 3 of 3 in 6.24 fps.

Name of file: P001_sag_57_1

Caption: Sagittal real-time spoiled GRE (FLASH2) of the stomach from participant P001 at t = 57 min. Slice 1 of 3 in 6.24 fps.

Name of file: P001_sag_57_2

Caption: Sagittal real-time spoiled GRE (FLASH2) of the stomach from participant P001 at t = 57 min. Slice 2 of 3 in 6.24 fps.

Name of file: P001_sag_57_3

Caption: Sagittal real-time spoiled GRE (FLASH2) of the stomach from participant P001 at t = 57 min. Slice 3 of 3 in 6.24 fps.

**In zipped folder: videos_P008**

Name of file: P001_cor_40_1

Caption: Coronal real-time spoiled GRE (FLASH2) of the stomach from participant P008 at t = 40 min. Slice 1 of 3 in 6.24 fps.

Name of file: P001_cor_40_2

Caption: Coronal real-time spoiled GRE (FLASH2) of the stomach from participant P008 at t = 40 min. Slice 2 of 3 in 6.24 fps.

Name of file: P001_cor_40_3

Caption: Coronal real-time spoiled GRE (FLASH2) of the stomach from participant P008 at t = 40 min. Slice 3 of 3 in 6.24 fps.

Name of file: P001_sag_43_1

Caption: Sagittal real-time spoiled GRE (FLASH2) of the stomach from participant P008 at t = 43 min. Slice 1 of 3 in 6.24 fps.

Name of file: P001_sag_43_2

Caption: Sagittal real-time spoiled GRE (FLASH2) of the stomach from participant P008 at t = 43 min. Slice 2 of 3 in 6.24 fps.

Name of file: P001_sag_43_3

Caption: Sagittal real-time spoiled GRE (FLASH2) of the stomach from participant P008 at t = 43 min. Slice 3 of 3 in 6.24 fps.

**In zipped folder: videos_P021**

Name of file: P021_cor_40_1

Caption: Coronal real-time spoiled GRE (FLASH2) of the stomach from participant P021 at t = 40 min. Slice 1 of 3 in 6.24 fps.

Name of file: P021_cor_40_2

Caption: Coronal real-time spoiled GRE (FLASH2) of the stomach from participant P021 at t = 40 min. Slice 2 of 3 in 6.24 fps.

Name of file: P021_cor_40_3

Caption: Coronal real-time spoiled GRE (FLASH2) of the stomach from participant P021 at t = 40 min. Slice 3 of 3 in 6.24 fps.

Name of file: P021_sag_43_1

Caption: Sagittal real-time spoiled GRE (FLASH2) of the stomach from participant P021 at t = 43 min. Slice 1 of 3 in 6.24 fps.

Name of file: P021_sag_43_2

Caption: Sagittal real-time spoiled GRE (FLASH2) of the stomach from participant P021 at t = 43 min. Slice 2 of 3 in 6.24 fps.

Name of file: P021_sag_43_3

Caption: Sagittal real-time spoiled GRE (FLASH2) of the stomach from participant P021 at t = 43 min. Slice 3 of 3 in 6.24 fps.
