# Supplementary material for: Quantitative Real‐Time MRI for the Assessment of Gastric Motility
Source: J Magn Reson Imaging. 2026 Feb 6;63(5):1482–95. doi: 10.1002/jmri.70243 (PMC13066535; doi:10.1002/jmri.70243)
Supplement: Supplementary file 1 — Data S1: jmri70243‐sup‐0001‐DataS1.zip. [file JMRI-63-1482-s001.zip › 20260112_Supplementary_Materials.docx]

# Supplementary Materials

# AI Training

All preprocessing, architecture selection, training, ensembling, thresholding and evaluation steps followed the default nnU-Netv2 pipeline without any manual modification. This includes the default learning-rate schedule, optimizer, loss function, data augmentation, patch sampling, 1000 epochs five-fold cross-validation on the images and the default binarization threshold of 0.5 for dice computation. For each task, nnU-Net automatically determined which architectures to train based on its internal model-planning rules. For *Volyntra* (transverse volumetric segmentation), nnU-Net generated three models (2D, 3D low-resolution and 3D full-resolution). For *Motiqva* (real-time sagittal segmentation), nnU-Net generated two models (2D and 3D full-resolution). When multiple architectures were available, nnU-Net formed an ensemble via voxel-wise averaging of the softmax probability maps, as per the default implementation. For *Volyntra*, transverse MR images of participants P001–P020 were loaded into OsiriX (version 14.1 Pixmeo SARL, Switzerland), and the gastric content was segmented manually at each time point using the brush tool (segmentation by LN, 2 yrs experience (exp), revision by LG, 5 yrs exp and WW, 35 yrs exp). The resulting 257 image-label pairs, one defined as all labeled frames of one sequence, were exported as NRRD files and integrated into the nnU-Net dataset structure. A dataset configuration file (dataset.json) was generated specifying imaging modality, label definitions, and metadata. For *Motiqva*, a total of 276 image-label pairs, one defined as all labeled frames of one sequence (and slice), were prepared for training. Data was obtained from 12 participants, each contributing four time points in the fasted state and four in the fed state. Instead of segmenting the entire sequence, only the initial 50 – 100 frames per time point were manually segmented to capture representative peristaltic movement.

Model training was performed on a dedicated university data-center node equipped with an NVIDIA RTX 4090 GPU (24 GB VRAM), a high-performance x86_64 server CPU, and 192 GB RAM. Coronal FLASH2 sequences were acquired but not included in the segmentation workflow.

# Dataset and Segmentation Results

For *Volyntra* three models were trained (2D, 3D low-resolution, 3D full-resolution). The best performance was achieved by an ensemble of the 3D low-resolution and 2D models, reaching a mean dice score of 0.939 on the cross-validation data. For *Motiqva*, 2D and 3D full-resolution nnU-Net models were trained. The best-performing model was an ensemble of both configurations, achieving a mean dice score of 0.922 on the cross-validation data.

# Computational Time

The initial preparation of the training dataset required manual segmentation of the stomach volume, resulting in 1 h for one subject (total: ~ 20 h) and the manual segmentation of the stomach, resulting in 15 min for 100 frames (total: ~ 17 h). Training of all *Volyntra* nnU-Net models took approximately 52 sec per epoch, while training of all *Motiqva* models took around 33 sec per epoch. The full analysis pipeline consisted of data conversion, preprocessing, automated segmentation and data extraction. Loading and preparing MRI data took around 30 min per subject. Both, motility and volume segmentation were performed in a total time of circa 3 h (for all measuring time points mentioned in the study protocol), including label output and the generation of video overlays. Checking the video outputs took 25 min per subject. Depending on how complex the correction was, the revision took several hours, with an average time of 10 sec per frame. Data analysis, including volume extraction, FFT-based peristaltic analysis and generating CSV output plots took 2 min for one subject (specifying data paths and distance factor between sagittal slices). Combining all steps, end-to-end analysis required approximately 5 – 6 hours per subject without counting the MRI measuring time. In a clinical context, where only a two-point determination was performed, this time was greatly reduced.

# Apparent Propagation Speed

To substantiate the interpretation of the apparent propagation speed used in the main manuscript, a time-delay based analysis was performed on a representative dataset (Subject P021, time point 43 min). The dominant gastric contraction frequency at this time point was 3 contractions per minute (0.05 Hz), consistent with physiological antral wave activity. Segmented area-time signals were extracted from sagittal FLASH2 acquisitions for the three slices. Signals were band-pass filtered around the dominant frequency band (0.03–0.07 Hz) to suppress respiratory and low-frequency drift components. Time delays (τ) between slices were estimated using normalized cross-correlation, with the lag corresponding to the maximum correlation coefficient taken as the propagation delay. Propagation speed was calculated as the quotient of anatomical distance and measured delay. Both border-to-border and center-to-center distance definitions were evaluated. Slice thickness was 5 mm and the subject-specific inter-slice distance was 10 mm.

| Slice Pair | Delay τ [s] | Border-to-Border Distance [mm] | Center-to-Center Distance [mm] | Speed Range [mm/s] |
| --- | --- | --- | --- | --- |
| Slice 1–2 | 4.97 | 10.0 | 15.0 | 2.01–3.02 |
| Slice 2–3 | 3.53 | 10.0 | 15.0 | 2.84–4.25 |
| Slice 1–3 | 8.49 | 25.0 | 30.0 | 2.94–3.53 |

Delay-based propagation speeds ranged from approximately 2 to 4 mm/s across slice pairs, in agreement with literature-reported antral propagation velocities. Differences arising from border-to-border versus center-to-center distance definitions remained within this physiological range. Using the frequency-distance approach applied in the main manuscript, the apparent propagation speed for this dataset was 1.75 mm/s. While numerically lower, this value is of the same order of magnitude as the delay-based estimates. The difference reflects that Eq. 3 yields an apparent propagation metric rather than a true phase velocity, due to broad spatial extent and partial-volume effects of the peristaltic wave.

Overall, the time-delay analysis supports that the simplified geometric approximation yields physiologically plausible propagation estimates and that the precise distance definition does not materially alter interpretation.


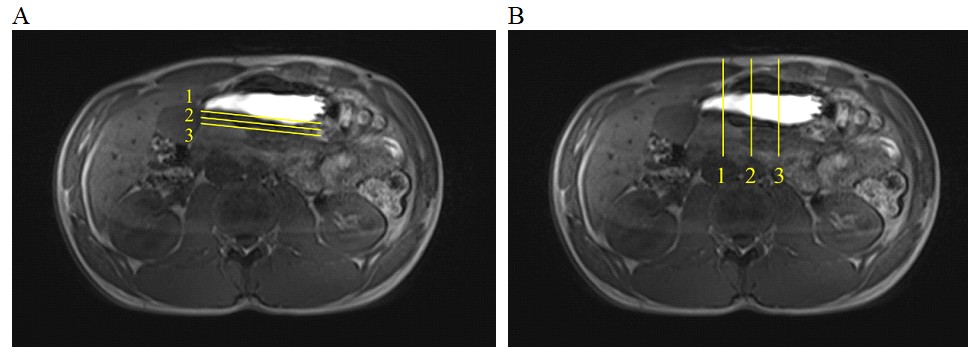


Figure S 1: Exemplary Representation of the Slice Placement in the Transverse VIBE Image (A: Coronal DF = 0 mm, B: Sagittal DF = individually from 7.5 – 17.5 mm).


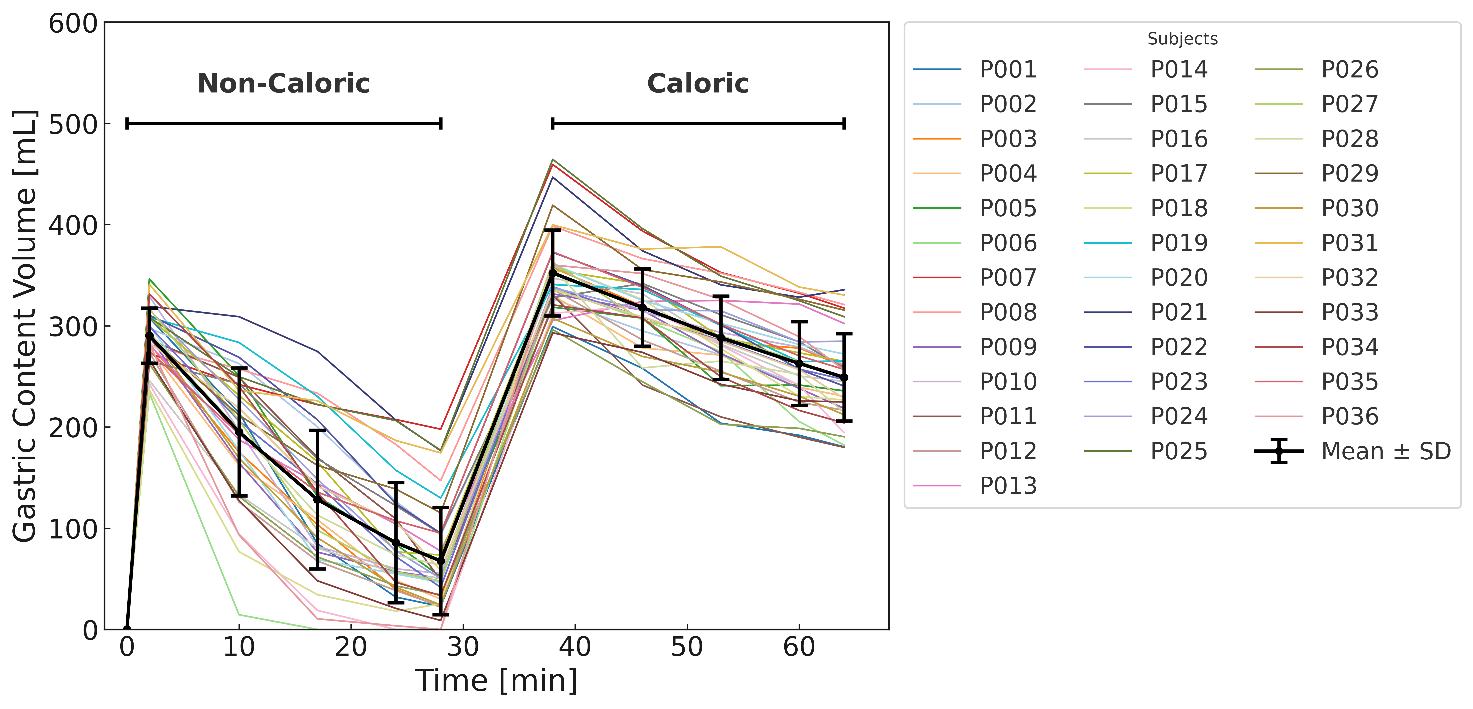


Figure S 2: Individual Gastric Content Volumes (GCV) P001 - P036, Mean GCV ± SD in Black over 64 min.

Table S 1: Number of Participants included for the Calculations of Frequency and Velocity for all Measurement Timepoints.

| Time (min) | Number of Subjects |
| --- | --- |
| 7 | 31 |
| 14 | 30 |
| 21 | 27 |
| 26.5 | 10 |
| 33.5 | 14 |
| 43 | 29 |
| 50 | 33 |
| 57 | 30 |
| 62.5 | 19 |

Table S 2: Data of all three Patients, Two-Point Determination for Volume at 11 and 46 min, Two-Point Determination for Peristalsis (Frequency, Velocity, Occlusion) at 18 and 38 min after Ingestion of a Calorie-Enriched Pineapple Juice at t = 0 min.

| Patient 1 | | | | | | |
| --- | --- | --- | --- | --- | --- | --- |
| Time (min) | Volume (mL) | Frequency (cpm) | Velocity (mm/s) | Occlusion (%) Slice 1 | Occlusion (%) Slice 2 | Occlusion (%) Slice 3 |
| 11 | 166.7 | - | - | - | - | - |
| 18 | - | 3 | 2.25 | 20.5 | 20.8 | 18.8 |
| 38 | - | 3 | 2.25 | 21.1 | 16.0 | 17.3 |
| 46 | 225.0 | - | - | - | - | - |
| Patient 2 | | | | | | |
| Time (min) | Volume (mL) | Frequency (cpm) | Velocity (mm/s) | Occlusion (%) Slice 1 | Occlusion (%) Slice 2 | Occlusion (%) Slice 3 |
| 11 | 274.2 | - | - | - | - | - |
| 18 | - | 3 | 1.5 | 27.7 | 30.4 | 25.7 |
| 38 | - | 3.3 | 1.7 | 52.2 | 54.7 | 57.0 |
| 46 | 263.5 | - | - | - | - | - |
| Patient 3 | | | | | | |
| Time (min) | Volume (mL) | Frequency (cpm) | Velocity (mm/s) | Occlusion (%) Slice 1 | Occlusion (%) Slice 2 | Occlusion (%) Slice 3 |
| 11 | 258.1 | - | - | - | - | - |
| 18 | - | 2.3 | 1.2 | 20.8 | 12.4 | 9.6 |
| 38 | - | 3 | 1.5 | 64.2 | 53.7 | 36.0 |
| 46 | 273.7 | - | - | - | - | - |
